# Long working hours and self-rated health in the national Brazilian working population: gender and employment status differences, 2019

**DOI:** 10.1186/s12889-023-16973-1

**Published:** 2023-10-25

**Authors:** Luciana de Melo Gomides, Mery Natali Silva Abreu, Ada Ávila Assunção

**Affiliations:** 1 Graduate Program in Public Health, Department of Preventive and Social Medicine, Federal University of Minas Gerais, Belo Horizonte, Brazil; 2 Institute of Pure and Applied Sciences. Health and Safety Engineering, University of Itajubá, Itabira, MG Brazil; 3 Department of Applied Nursing, Federal University of Minas Gerais, Belo Horizonte, MG Brazil

**Keywords:** Long working hours, Employment status, Self-rated health, Gender, Brazil

## Abstract

**Background:**

The regulation of working hours is governed by legal standards in formal employment. While the association between long working hours and various health outcomes has been extensively studied, there is limited evidence regarding Brazil. The objective of this study was to investigate the association among working hours, employment status, and self-rated health (SRH), taking into account differences between men and women in a national representative sample of the working population in Brazil.

**Methods:**

A cross-sectional study was conducted among a representative sample of 33,713 workers in Brazil to assess self-rated health (SRH). We examined the associations between working hours and employment status, categorizing working hours as standard (40–44 h per week) or long (> 44 h per week), and employment status as formal or informal. Logistic regression models were employed, adjusting for sociodemographic, occupational characteristics, and health behaviors. Probabilities of negative SRH were calculated for men and women in different exposure profiles. Results were stratified by gender, and 95% confidence intervals (CIs) were applied to the findings.

**Results:**

The prevalence of long working hours was higher among informal workers for both men and women. Adjusted odds ratio (AOR) results revealed that informal employment (AORwomen = 1.53; 95% CI: 1.13–2.07 and AORmen = 1.55; 95% CI: 1.22–1.96) and long working hours (AORwomen = 1.23; 95% CI: 1.06–1.43 and AORmen = 1.14; 95% CI: 1.00-1.30) were independently associated with negative SRH. Significant interactions between long working hours and informal employment were observed. Among individuals with the same exposure profile, women who engaged in long working hours had a higher probability of reporting negative SRH compared to men.

**Conclusions:**

The results of this study are in line with the literature, as differences between men and women in the likelihood of negative self-rated health were observed. The adverse health effects underscore the importance of implementing intersectoral actions to inform the revision of regulations concerning weekly working hours and the expansion of informal employment in low- and middle-income countries.

## Background

Long working hours are considered the main occupational risk for estimated deaths in Latin American and Caribbean countries [1]. Excessive working hours result in chronic fatigue and occupational stress, which can have negative impacts on sleep duration and quality [1]. Both factors are associated with unhealthy behaviors such as excessive alcohol and tobacco use, low adherence to physical activity during leisure time, and an imbalanced diet. These behaviors are considered mediators in the chain linking exposure to long working hours and poorer health outcomes [2]. The increased risk of cardiovascular diseases [3], neurovascular conditions [2], diabetes [2], depression [4], and other chronic illnesses in groups exposed to long working hours has been well documented.

The maximum weekly working hours allowed by the Brazilian Federal Constitution of 1988 is 40 h, with a limit of 44 h per week [5]. The flexibilization of labor relations regulated in Brazil by Law no. 13.467, dated July 13, 2017 [6] and known as the Labor Reform, modified the legal framework that governed labor relations. New definitions under the aforementioned law allowed, for example, an increase in the number of overtime hours worked. Furthermore, the limit for part-time work increased from 25 to 30 h per week [7].

This liberalizing reform of labor regulation came amid an economic crisis that began in 2015. In 2017, a decrease of 26.9% in the gross domestic product (GDP) was recorded compared to 2014 [8]. Since 2015, there has been a continuous increase in the number of jobs without formal contracts. In 2019, the unemployment rate reached 11.7% of the economically active population, nearly 5% points higher than the rate observed in 2014 (6.9%) [9].

The structural inequalities in the labor market, exemplified by high unemployment rates and a significant proportion of informal labor, among other characteristics, have been exacerbated by the transformations brought about by productive restructuring. In recent decades, the proportion of workers without regular employment or in precarious work arrangements has been quite high [10]. In an environment of intense competition among markets, the labor reform of 2017 [6] aimed to reduce labor costs. This new dynamic of capital accumulation encouraged the connection between formal and informal employment relationships, such as through temporary service companies or small production units on the fringes of the main productive core. The variety of occupational situations and worker mobility between well-structured segments and precarious segments constitute the current Brazilian labor market. The regulation of job instability and wage reduction, along with increased work intensity, has altered work environments already transformed by technological innovations [11], with consequences of varying magnitude on workers’ health.

In Brazil, informal employment assumes a particularly considerable prevalence, representing a substantial portion of the workforce. This reality, common in several countries, is even more pronounced in the Brazilian context, where the informal employment rate is especially high [12]. It is pertinent to note that individuals engaged in informal occupations, in contrast to those formally linked to employment, lack comprehensive labor regulations such as employment protection laws, the establishment of a minimum wage, and social security contributions [13].

The consideration of the link between sex and gender has been suggested by authors studying the intersections of work and health [14]. Sex pertains to biological factors, including chromosomal and hormonal aspects. Gender, on the other hand, encompasses socially constructed expectations and roles deemed appropriate for either men or women [15]. It encompasses social differences in attributes and opportunities associated with femininity or masculinity. This type of role assignment shapes experiences and inequalities in functions, activities, access to resources, and opportunities. For instance, the distribution of time allocated to work and domestic tasks remain imbalanced between men and women, despite the increased participation of women in the labor market. Stratified analysis by sex provides a pathway for incorporating gender perspectives in the interpretation of any identified differences. In this context, it helps avoid narrow or limited conclusions.

In this article, we comparatively assessed working hours based on findings from the literature. The hypothesis in this study relates to the higher likelihood of negative self-rated health (SRH) among workers with informal employment contracts and among those working more than 44 h per week. The analyses were stratified by sex to examine the second hypothesis, which pertains to gender norms in time use. It is known that the distribution of time dedicated to work, household chores, and leisure activities remains unbalanced between men and women [16–18].

Employment relationships are recognized as determinants that influence the duration of exposure to stressors and other known occupational risks [19]. From this perspective, it is relevant to examine employment status (formal or informal) when addressing the hypothesis concerning negative SRH among workers exposed to long working hours. Therefore, interaction models were developed.

We did not find any national studies that have focused on the health status of the workforce, nor are there representative results for the Brazilian population regarding employment or work characteristics that would enable a discussion of the aforementioned hypotheses.

Considering the cross-sectional design of the National Health Survey, the data source for our study, we decided to investigate the prevalence of negative self-perceived health to address the health dimension mentioned in our hypotheses. SRH is a consistent measure of workforce health status [20]. By means of a single question—“In general, how would you rate your health?“—it is possible to assess an individual’s subjective perception of their own health [21]. Frequently incorporated in population surveys, this validated inquiry is regarded as a consistent health indicator. The objective of this study was to investigate the association among working hours, employment status, and self-rated health (SRH), taking into account differences between men and women in a national representative sample of the working population in Brazil.

## Methods

### Study design and sample

This was a cross-sectional study based on secondary data collected from the Brazilian National Health Survey (NHS) of 2019. The NHS is a nationwide household survey conducted by the Brazilian Institute of Geography and Statistics (IBGE) in partnership with the Ministry of Health [22]. The NHS sample is probabilistic and representative of the Brazilian population aged 15 years and older. The sampling process used random and cluster sampling, divided into three stages: (i) census tracts; (ii) households; and (iii) one resident from each household. The sample size estimation was guided by some indicators from the previous edition, the NHS of 2013. A total of 108,525 households were selected. The final sample consisted of 90,846 interviews, with a response rate of 96.5%. Detailed information on the sampling and data collection has been published previously [23]. For the purpose of this study, participants from the NHS sample who responded to the individual questionnaire, were aged 18 years or older, and worked a weekly schedule of 40 h or more were selected. Unemployed participants, students, military personnel, employers, individuals engaged in domestic work at home or for close relatives, individuals of working age who had given up seeking employment, and adults permanently out of the labor market due to incapacity were excluded.

### Dependent variable: self-rated health (SRH)

SRH is considered a consistent measure of workforce health [24]. This variable was derived from the response to the question “In general, how do you assess your health?“. Response options were grouped into a dichotomous indicator, with the study outcome being associated with the negative SRH category. This category encompasses responses such as “fair,“ “poor,“ and “very poor.“ In contrast, the “positive health assessment” category includes responses indicating “very good” and “good” health.

### Independent variables: working hours and employment status

Working hours were determined according to the regulation of working hours in Brazil [5], which establishes a duration of 40 to 44 hours. The questions E17. “How many hours do you normally work per week in this job?” and E19. “How many hours do you normally work per week in other job(s)?“ were used. The responses to the following questions were summed and categorized into two categories: 1. regular working hours, 40 to 44 hours per week; and 2. long working hours, more than 44 hours per week.

The variable consisted of two categories, informal employment and formal employment, constructed from the response to closed-ended questions in the questionnaire. Informal employees were defined as follows: domestic workers; public or private employees without statutory employment and without a work permit; self-employed individuals; and unpaid workers providing assistance to a household member. Formal employees encompassed public and private employees with a signed work permit.

### Covariates

Three groups of adjustment variables included sociodemographic, occupational, and health behavior characteristics. The first group included age group (18–24 years, 25–54 years, and 55 years or more), education level (less than primary, primary, secondary, higher education), marital status (living alone, not alone), region of residence (South, Southeast, North, Northeast, and Midwest), and per capita income in minimum wages (< 1 wage, 1⊢3 wages, 3⊢5 wages, and ≥ 5 wages). The last variable corresponded to the reference minimum wage in 2019 (US$203). Self-reported skin color/race was categorized as white and nonwhite (including black, mixed, Asian, and indigenous individuals). The second group included multiple jobs (yes, no), work-related accidents (yes, no) and occupational risk exposure, encompassing exposure to chemical, physical, or biological hazards at work (yes, no). The third group included covariates related to health behaviors. These covariates were assessed based on the following items: drinking (yes, no), smoking (yes, no), and engagement in physical activity (yes, no or no answer). Regarding physical activity, the guidelines set by the World Health Organization [25] were followed, where “yes” indicates engaging in at least 150 min of moderate physical activity per week or 75 min of vigorous physical activity per week, and “no” indicates engaging in less than 150 min of moderate physical activity per week or less than 75 min of vigorous physical activity per week.

### Statistical analysis

Data analysis was performed using Stata® 16.0 statistical software (StataCorp LP, College Station, Texas, USA). The appropriate weights derived from the complex sampling design were taken into account in all analyses using the survey command with the svy prefix. Descriptive analyses of the sample were performed for all study variables, presenting weighted prevalence estimates and their respective 95% confidence intervals (CIs). All analyses were stratified by sex. Pearson’s chi-square test was used to compare differences between groups, with a significance level set at p < 0.05.

The association among working hours, employment status, and negative SRH was assessed through crude and adjusted logistic regression analyses, providing measures of association, i.e., odds ratios (ORs). Furthermore, variables that obtained a value of p < 0.20 in the crude model were considered to obtain the adjusted model.

In the adjusted analysis, a hierarchical model of determination [26] was used. This type of analysis considered the effect of each variable on the outcome while controlling for possible confounding effects between proximal and distal variables. Individual characteristics (age and self-reported skin color or race) were considered distal variables that could act as determinants of sociodemographic and occupational status (marital status, education, per capita income, region of residence, occupational accidents, and exposure to occupational risks), which in turn could influence behavior and lifestyle habits (smoking, alcoholism, and level of physical activity), ultimately contributing to negative SRH. The independent variables (working hours and employment status) were simultaneously entered into the final model: Model 1. The backward method was used for variable selection in each group, retaining only those variables significant at the 5% level.

The effect of the interaction between working hours and employment status on negative SRH was investigated in Model 3 (Model 1 + interaction). The magnitude of the association was estimated using adjusted odds ratios (AOR) and their respective 95% CIs at all stages of analysis. Model fit was assessed using the Hosmer–Lemeshow test.

The interaction effect was approached from two different perspectives. The exact moderating effects of working hours and employment status on each other were examined through stratified regression analyses. The first analysis tested the association of employment status with negative SRH within each category of working hours. In the second analysis, the association between working hours and negative SRH was analyzed by employment status.

After defining the adjusted models, predictive calculations were conducted by work shift category for both men and women, following two distinct profiles: the first composed of characteristics associated with a higher likelihood of negative SRH, and the second with characteristics associated with a lower likelihood.

## Results

### Characteristics of the study participants

A total of 33,713 interviews were eligible for analysis. Descriptive characteristics of the sample and estimated adult population are presented in Table 1. The majority of workers were male (61.2%; 95% CI: 60.2–62.1), aged 25 to 54 years (72.2%; 95% CI: 71.3–73.0), resided in the Southeast region (46.5%; 95% CI: 45.3–47.8), were nonwhite (55.3%; 95% CI: 54.3–56.3), had a secondary education (41.0%; 95% CI: 40.1–41.9) and had a per capita income below 3 minimum wages. Regarding health-related behaviors, the majority of workers reported alcohol consumption (71.8%; 95% CI: 70.9–72.8), lack of physical activity (66.3%; 95% CI: 65.4–67.2), and no history of smoking (87.0; 95% CI: 86.3–87.6). The estimated negative SRH in the country was 24.5% (95% CI: 23.7–25.3). One-third of the sample reported working more than 44 h per week (33.1; 95% CI: 32.1–34.0). The majority were engaged in formal employment (58.0%; 95% CI: 57.0–59.0) with a single job (94.5%; 95% CI: 94.1–95.0).

**Table 1:** Description of the study population (n = 33,713) by sex in 2019, NHS, Brazil

		Total sample		Women		Men	
		**n**	**%w (95%CI)**	**n**	**%w (95%CI)**	**n**	**%w (95%CI)**
Self-rated health	Positive	24,512	75.5 (74.7–76.3)	8951	73.00 (71.6–74.3)*	15,561	77.1(76.0-78.2)
	Negative	9201	24.5 (23.7–25.3)	3822	27.1 (25.7–28.4)	5379	22.9 (21.8–25.3)
Sociodemographic							
Sex	women	12,773	38.9 (37.9–39.8)	12,773	100		
	men	20,940	61.2 (60.2–62.1)			20,940	100
Age group (years)	18 a 24	2806	13.1 (12.3–14.0)	1059	13.2 (11.9–14.6)*	1747	13.1 (12.1–14.1)
	25 a 54	25,233	72.2 (71.3–73.0)	9891	74.2 (72.7–75.6)	15,342	70.9 (69.7–72.0)
	≥ 55	5674	14.7 (14.1–15.4)	1823	12.6 (11.8–13.6)	3851	16.1 (15.3–16.9)
Skin color/race	White	12,752	44.7 (43.7–45.7)	5118	47.7 (46.2–49.3)*	7634	42.8 (41.6–44.1)
	Nonwhite^τ^	20,961	55.3 (54.3–56.3)	7655	52.3 (50.7–53.8)	13,306	57.2 (56.0-58.4)
Education level	Higher education	6743	19.7 (18.8–20.7)	3652	26.8 (25.3–28.3)*	3091	15.3 (14.2–16.4)
	Secondary	12,479	41.0 (40.1–41.9)	5245	44.4 (42.9–46.0)	7234	38.8 (37.6–40.1)
	Primary	4900	15.1 (14.4–15.8)	1538	12.2 (11.2–13.3)	3362	17.0 (16.1–17.9)
	Less than primary	9591	24.2 (23.3–25.0)	2338	16.6 (15.5–17.8)	7253	28.9 (27.8–30.1)
Marital status	Not alone	22,374	69.2 (68.2–70.2)	7033	60.1 (58.5–61.6)*	15,341	75.0 (73.8–76.3)
	Living alone	11,339	30.8 (29.8–31.8)	5740	40.0 (38.4–41.5)	5599	25.0 (23.8–26.2)
Region of residence	South	5001	16.8 (16.1–17.5)	2023	17.5 (16.5–18.5)*	2978	16.3 (15.4–17.3)
	North	6254	6.9 (6.5–7.3)	2195	6.2 (5.8–6.6)	4059	7.3 (6.8–7.8)
	Southeast	8108	46.5 (45.3–47.8)	3194	48.0 (46.4–49.6)	4914	45.6 (43.9–47.3)
	Midwest	4643	8.4 (8.0-8.9)	1793	8.3 (7.7–8.8)	2850	8.5 (8.0-9.1)
	Northeast	9707	21.4 (20.7–22.3)	3568	20.1 (19.1–21.2)	6139	22.3 (21.3–23.3)
Per capita income (MW)^ττ^	≥ 5	2177	5.4 (4.9–5.9)	943	5.9 (5.3–6.6)*	1234	5.0 (4.4–5.6)
	≥ 3 to 5	2499	7.1 (5.6–7.6)	1075	8.0 (7.1-9.0)	1424	6.5 (5.9–7.1)
	≥ 1 to 3	14,763	46.9 (45.9–48.0)	5831	49.5 (48.0–51.0)	8932	45.3 (44.0-46.6)
	< 1	14,274	40.6 (39.6–41.7)	4924	36.6 (35.1–38.1)	9350	43.2 (42.0-44.5)
Health behaviors							
Smoking	No	29,169	87.0 (86.3–87.6)	11,611	91.5 (90.8–92.2)*	17,558	84.1 (83.1–85.0)
	Yes	4544	13.1 (12.4–13.7)	1162	8.5 (7.8–9.3)	3382	15.9 (15.0-16.9)
Drinking	No	8424	28.2 (27.3–29.1)	3253	28.7 (27.3–30.2)***	5171	27.8 (26.7–29.0)
	Yes	25,289	71.8 (70.9–72.8)	9520	71.3 (69.8–72.7)	15,769	72.2 (71.0-73.3)
Physical activity	No	22,596	66.3 (65.4–67.2)	3862	70.0(68.6–71.3)*	6649	64.0 (62.8–65.2)
	Yes	10,511	31.5 (30.6–32.4)	8748	28.5 (27.2–29.8)*)	13,848	33.4 (32.3–34.7)
	NA	606	2.2 (1.9–2.5)	163	1.6 (1.2-2.0)	443	2.6 (2.2-3.0)
Occupational							
Employment status	Formal	17,772	58.0 (57.0–59.0)	7604	63.1 (61.6–64.5)*	10,168	54.8 (53.5–56.1)
	Informal	15,941	42.0 (41.0–43.0)	5169	37.0 (35.5–38.5)	10,772	45.2 (43.9–46.5)
Working hours	40–44 h/week	22,398	67.0 (66.0-67.9)	8957	71.3 (69.9–72.6)*	13,441	64.2 (63.0-65.5)
	> 44 h/week	11,315	33.1 (32.1–34.0)	3816	28.8 (27.4–30.1)	7499	35.8 (34.5–37.0)
Multiple jobs	No	31,705	94.5 (94.1–95.0)	11,923	94.0 (93.3–94.7)	19,782	94.8 (94.2–95.4)
	Yes	2008	5.5 (5.1-6.0)	850	6.0 (5.3–6.7)	1158	5.2 (4.6–5.8)
Occupational risks	No	16,362	48.0 (46.9–49.0)	8305	63.5 (62.0–65.0)*	17,803	38.1 (36.8–39.3)
	Yes	17,351	52.1 (51.0-53.1)	4468	36.5 (35.0–38.0)	3137	61.9 (60.7–63.2)
Work accident	No	32,674	96.8 (96.5–97.2)	12,503	97.8 (97.2–98.2)*	8057	96.3 (95.7–96.8)
	Yes	1039	3.2 (2.8–3.6)	270	2.3 (1.8–2.8)	12,883	3.7 (3.3–4.3)

%w: weighted frequency. 95% CI: Confidence intervals of 95%; NA: No answer

Rao-Scott χ2 test p value for the comparison between genders *p < 0.001; **p < 0.05; ***p > 0.05

^τ^Nonwhite: black, mixed, Asian and indigenous. ^ττ^Minimum wage in 2019 (SM) in 2019 US$203.

Compared to men, in general, women in Brazil had higher levels of education, reported a higher per capita income, had a lower percentage of individuals living alone, were less engaged in more physical activity and a higher percentage of negative SRH. Men had a higher percentage of individuals aged 55 years or older, were nonwhite, reported smoking, were more exposed to long working hours, had a higher proportion of informal employment, and had higher rates of occupational risks and work-related accidents than women (Table 1).

### Prevalence of long working hours

Table 2 presents the results of the comparative analysis between the duration of working hours (standard and long). The prevalence of long working hours was higher among men (35.8%; 95% CI: 34.5–37.0) than among women (28.8%; 95% CI: 27.4–30.1).

**Table 2:** Description of the study population (N = 33,713) according to working hours by sex in 2019, NHS, Brazil

		Working hours				
		**Women**			**Men**	
		**Standard** **(40–44 h/week)**	**Long** **(> 40 h/week)**		**Standard** **(40–44 h/week)**	**Long** **(> 40 h/week)**
		**%w (95% CI)**	**%w (95% CI)**		**%w (95% CI)**	**%w (95% CI)**
Self-rated health	Positive	75.1 (73.5–76.6)		67.6 (65.1–70.1)	78.3 (77.1–79.4)	75.1 (72.8–77.2)
	Negative	24.9 (23.4–26.5)		32.4 (29.9–35.0)	21.8 (20.6–22.9)	24.9 (22.8–27.2)
Sociodemographic						
Age group (years)	18 a 24	13.6 (12.0-15.3)		12.1(10.1–14.4)	13.8 (12.5–15.1)	11.9 (10.4–13.5)
	25 a 54	74.5 (72.7–76.2)		73.5 (70.9–75.9)	70.4 (69.0-71.8)	71.6 (69.6–73.5)
	≥ 55	11.9 (10.9–13.0)		14.4 (12.6–16.4)	15.8 (14.8–16.9)	16.6 (15.2–18.1)
Skin color/race	White	49.2 (47.3–51.1)		44.2 (41.5–46.8)	43.2 (41.7–44.8)	42.2 (39.8–44.6)
	Nonwhite^τ^	50.8 (48.9–52.7)		55.9 (53.2–58.5)	56.8 (55.3–58.4)	57.8 (55.4–60.2)
Education level	Higher education	29.4 (27.7–31.3)		20.2 (18.1–22.5)	16.3 (15.1–17.7)	13.3 (11.9–14.9)
	Secondary	44.8 (42.8–46.7)		43.5 (40.8–46.2)	39.8 (38.2–41.4)	37.2 (35.3–39.0)
	Primary	10.6 (9.6–11.8)		16.1 (13.9–18.5)	16.2 (15.1–17.3)	18.4 (16.9–20.1)
	Less than primary	15.2 (14.0-16.4)		20.3 (18.1–22.6)	27.7 (26.4–29.1)	31.1 (29.2–33.0)
Marital status	Not alone	60.1 (58.2–61.9)		60.0 (57.2–62.7)	72.7 (71.2–74.2)	79.2 (77.4–80.9)
	Living alone	39.9 (38.1–41.8)		40.0 (37.3–42.8)	27.3 (25.8–28.8)	20.8 (19.1–22.6)
Region of residence	South	6.4 (5.9-7.0)		5.7 (5.0-6.4)	7.9 (7.2–8.5)	6.3 (5.7–6.9)
	North	18.5 (17.3–19.7)		15.0 (13.5–16.7)	17.3 (16.2–18.5)	14.5 (13.3–15.7)
	Southeast	47.7 (45.8–49.5)		48.7 (46.0-51.3)	43.7 (41.9–45.4)	49.1 (46.6–51.5)
	Midwest	8.8 (8.2–9.5)		6.8 (6.0-7.7)	9.1 (8.4–9.8)	7.6 (6.8–8.4)
	Northeast	18.6 (17.5–19.8)		23.9 (22.0-25.8)	22.1 (21.0-23.3)	22.6 (21.1–24.1)
Per capita income (MW)^ττ^	≥ 5	5.5 (4.9–6.3)		6.9 (5.8–8.2)	4.4 (3.8-5.0)	6.1 (5.2–7.2)
	≥ 3 to 5	8.2 (7.2–9.3)		7.5 (6.1–9.1)	6.5 (5.9–7.2)	6.5 (5.6–7.5)
	≥ 1 to 3	51.0 (49.2–52.8)		45.9 (43.2–48.6)	45.7 (44.3–47.2)	44.5 (42.4–46.6)
	< 1	35.3 (33.5–37.0)		39.8 (37.1–42.5)	43.4 (41.9–44.9)	43.0 (41.0–45.0)
Health behavior						
Smoking	No	91.8 (90.7–92.7)		90.8 (89.3–92.2)	84.1 (82.9–85.3)	83.9 (82.5–85.3)
	Yes	8.3 (7.3–9.3)		9.2 (7.8–10.7)	15.8 (14.7–17.1)	16.1 (14.7–17.5)
Drinking	No	28.5 (26.8–30.2)		29.3 (26.9–31.8)	27.7 (26.4–29.1)	28.1 (26.1–30.1)
	Yes	71.5 (69.8–73.2)		70.7 (68.2–73.1)	72.3 (70.9–73.7)	72.0 (69.9–73.9)
Physical activity	No	29.1 (27.5–30.7)		27.0 (24.7–29.3)	34.6 (33.1–36.1)	31.4 (29.7–33.2)
	Yes	69.5 (67.8–71.1)		71.3 (68.9–73.6)	63.0 (61.5–64.5)	65.7 (63.9–67.6)
	NA	1.5 (1.1-2.0)		1.8 (1.0–3.0)	2.4 (2.0-2.9)	2.9 (2.2–3.6)
Occupational						
Employment status	Formal	68.5 (66.8–70.1)		49.6 (46.8–52.4)	59.0 (57.5–60.6)	47.3 (45.0-49.5)
	Informal	31.5 (29.9–33.2)		50.4 (47.6–53.2)	41.0 (39.4–42.5)	52.8 (50.5–55.0)
Multiple jobs	No	98.0 (97.4–98.4)		84.2 (82.2–86.1)	99.1 (98.7–99.3)	87.2 (85.7–88.6)
	Yes	2.0 (1.6–2.6)		15.8 (13.9–17.8)	0.9 (0.7–1.3)	12.8 (11.4–14.3)
Occupational risks	No	66.0 (64.3–67.7)		57.3 (54.4–60.3)	38.9 (37.2–40.5)	36.7 (34.5–38.9)
	Yes	34.0 (32.4–35.7)		42.7 (39.8–45.6)	61.2 (59.5–62.8)	63.3 (61.1–65.5)
Work accidents	No	97.8 (97.1–98.3)		97.7 (97.0-98.3)	96.7 (96.1–97.1)	95.5 (94.3–96.5)
	Yes	2.2 (1.7–2.9)		2.3 (1.7-3.0)	3.3 (2.9–3.9)	4.5 (3.5–5.7)
Total		**71.3 (69.9–72.6)**		**28.8 (27.4–30.1)**	**64.2 (63.0-65.5)**	**35.8 (34.5–37.0)**

%w: weighted frequency. 95% CI: Confidence intervals of 95%; NA: No answer

^τ^Nonwhite: black, mixed, Asian and indigenous. ^ττ^Minimum wage in 2019 (SM) in 2019 US$203.

When compared to workers who work between 40 and 44 h per week, those who long working hours showed a higher percentage of individuals with low levels of education, informal employment contracts, multiple jobs, and SRH negative health, both for the group of men and women (Table 2).

Notably, significant differences between men and women were observed among the groups with long working hours. Our study revealed that in this group of workers who work more than 44 h per week, there is a higher proportion of men reporting less than primary education, not living alone, being smokers, exposed to occupational risks, and experiencing work-related accidents than women. However, among female workers, higher levels of education and a higher proportion of individuals who engaged in physical activity and had negative SRH were observed. No differences were found in terms of age group, skin color/race, per capita income, or employment status, as presented in Table 2.

### Analyses of working hours, employment status, and SRH

According to the results of the unadjusted analysis, all variables achieved significance (p < 0.20), except for the multiple jobs. In the final multivariable model (Model 1), the following variables remained associated with negative SRH for both men and women: age group, education level, region of residence, physical activity, per capita income, and employment status. Exposure to occupational risks and work-related accidents remained associated with negative SRH in the women’s group, while skin color/race and drinking were significantly associated with the outcome variable among men.

In adjusted Model 1 (Table 3), working more than 44 h per week (AOR_women_=1.23; 95% CI: 1.06–1.43 and AOR_men_=1.14; 95% CI: 1.00-1.30) and informal employment (AOR_women_=1.53; 95% CI: 1.13–2.07 and AOR_men_=1.55; 95% CI: 1.22–1.96) were independently associated with negative SRH for both men and women.

**Table 3:** Associations between working hours, employment status and self-rated health (SRH) adjusted for covariates by sex, 2019, NHS, Brazil

Model		Negative self-reported health					
		Women			Men		
		OR	95%CI	p value	OR	95%CI	p value
Model 1	**Working hours**						
	40 a 44 h/week	1.00			1.00		
	> 44 h/week	1.23	1.06–1.43	0.008	1.14	1.00-1.30	0.0480
	**Employment status**						
	Formal	1.00			1.00		
	Informal	1.18	1.02–1.36	0.028	1.15	1.03–1.28	0.016
	*Hosmer–Lemeshow test*	0.660			0.860		
Model 2	**Working hour x employment status**	1.53	1.13–2.07	0.006	1.55	1.22–1.96	< 0.001
	*Hosmer–Lemeshow test*	0.989			0.999		

Model 1 women: regression adjusted for age group, education level, region of residence, per capita income, physical activity, occupational risk exposure, and work accidents. Model 1 men: regression adjusted for age group, skin color/race, education level, region of residence, per capita income, drinking, and physical activity. Model 2 introduced the interaction term of working hours x employment status based on Model 1 for both genders; only the interaction coefficient is shown in Table 2. 95% confidence interval (CI95%).

To assess the interaction effect, we produced Model 2 by incorporating a working hour versus employment status factor into Model 1. The interaction coefficient showed statistical significance for both men and women (AOR_women_=1.53; 95% CI: 1.13–2.07 and AOR_men_=1.55; 95% CI: 1.22–1.96) (Table 3).

According to the interaction test, the association between employment status and negative SRH was dependent on the duration of weekly working hours. Among women who reported working more than 44 h per week, informal employment was associated with negative SRH (AOR_women_=1.59; 95% CI: 1.23–2.06). A similar trend was observed among men who worked long hours, where informal employment was associated with negative SRH (AOR_men_=1.50; 95% CI: 1.24–1.81). For groups with regular working hours, informal employment was not associated with negative SRH, although the results did not reach statistical significance for either men or women (Table 4).

**Table 4:** Analysis of self-rated health and employment status moderated by working hours. 2019, NHS, Brazil

Employment status	Negative self-reported health							
	Women				Men			
	Working hours 40 a 44 h/week		Working hours >44 h/week		Working hours 40 a 44 h/week		Working hours >44 h/week	
	**OR**	**95%CI**	**OR**	**95%CI**	**OR**	**95%CI**	**OR**	**95%CI**
Formal	1.00		1.00		1.00		1.00	
Informal	1.01	0.85–1.20	1.59	1.23–2.06	0.98	0.85–1.14	1.50	1.24–1.81
*Hosmer–Lemeshow test*	0.911		0.979		0.928		0.250	

Women: Regression model adjusted for age group, education level, region of residence, per capita income, physical activity, occupational risk exposure, and work accidents stratified by working hours. Men: Regression model adjusted for age group, skin color/race, education level, region of residence, per capita income, drinking, and physical activity stratified by working hours. 95% confidence interval (CI95%).

To evaluate the association between working hours and SRH according to employment status, a specific stratified regression analysis was conducted. The results indicated that for participants with informal employment, working more than 44 h per week was associated with negative SRH (AOR_women_=1.54; 95% CI: 1.25–1.91 and AOR_men_=1.43; 95% CI: 1.20–1.70). No associations were observed between long working hours and negative SRH among workers with formal employment. However, these results did not reach statistical significance (Table 5).

**Table 5:** Analysis of self-rated health and working hours moderated by employment status. 2019, NHS, Brazil

Working hours	Negative self-reported health							
	Women				Men			
	Formal		Informal		Formal		Informal	
	**OR**	**95%CI**	**OR**	**95%CI**	**OR**	**95%CI**	**OR**	**95%CI**
40 a 44 h/week	1.00		1.00		1.00		1.00	
> 44 h/week	1.00	0.81–1.24	1.54	1.25–1.91	0.90	0.74–1.09	1.43	1.20–1.70
*Hosmer–Lemeshow test*	0.877				0.973		0.917	

Women: Regression model adjusted for age group, education level, region of residence, per capita income, physical activity, occupational risk exposure, and work accidents stratified by employment status. Men: Regression model adjusted for age group, skin color/race, education level, region of residence, per capita income, drinking, and physical activity stratified by employment status. 95% confidence interval (CI95%).

In Profile 1, the probability outcome for the disadvantaged female group in SRH included informal employment, age equal to or older than 55 years, no formal education, residents of the North region, per capita income equal to or less than 1 minimum wage, self-reported work accidents and occupational risk exposure, and no physical activity. In contrast, Profile 2 included formal employment, age equal to or less than 18 years, higher educational attainment, residents of the South region, per capita income equal to or greater than 5 minimum wages, no self-reported work accidents and occupational exposure, and physical activity. The probabilities of negative SRH found for those who have regulated working hours were 88.13% (95% CI: 82.00-94.30) for Profile 1 and 4.46% (95% CI: 2.55–6.36) for Profile 2. Meanwhile, for those with long working hours, the probabilities were 90.11% (95% CI: 84.88–95.33) for Profile 1 and 5.41% (95% CI: 3.13–7.69) for Profile 2.

In the men’s group, Profile 1 included informal employment, age equal to or older than 55 years, nonwhite, no formal education, residents of the North region, per capita income equal to or less than 1 minimum wage, self-reported alcohol consumption, and no physical activity. For Profile 2: formal employment, age equal to or less than 18 years, higher educational attainment, residents of the South region, per capita income equal to or greater than 5 minimum wages, no self-reported alcohol consumption, and physical activity. The probabilities of negative SRH found for those with regulated working hours were 59.42% (95% CI: 54.65–64.20) for Profile 1 and 2.03% (95% CI: 1.17–2.90) for Profile 2. Meanwhile, for those with long working hours, the probabilities were 62.58% (95% CI: 57.92–67.25) for Profile 1 and 2.32% (95% CI: 1.37–3.26) for Profile 2.

## Discussion

To our knowledge, this is the first nationwide study to explore hypotheses regarding the likelihood of negative health, assessed through the question about SRH, when workers are exposed to long working hours [20]. As expected, [1, 7], this likelihood was higher in the portion of the workforce, regardless of whether men and women were engaged in informal employment. Differences between groups were addressed to confirm both the longer duration of working hours among men [17, 18] and the higher prevalence of negative SRH among women [27]. The association between long working hours and informal employment confirmed our hypotheses, as discussed below [28].

The increased likelihood of negative SRH among those exposed to long working hours was expected [2, 29]. The longer the duration of the working hours, the more time an individual is exposed to occupational risks and the less time they have for rest and recovery, resulting in physical and mental overload. These situations contribute to disruptions in the body, which, in the long term, can lead to various chronic diseases [2, 3, 30, 31].

Based on the results, which involved stratifying the analysis by gender to explore the hypothesis, important observations were made. Our findings indicated that the percentage of men working long hours is higher than that of women. Notably, among those working long hours, women exhibited a higher prevalence of negative SRH than men. When assessing the odds of having negative SRH, it became evident that women, regardless of their working hours, had a higher likelihood of reporting negative SRH than men. Specifically, female informal workers working long hours had the highest probability, exceeding 90%, while male informal workers in the same profile had a probability of approximately 60%.

The interpretation of the results on negative SRH is possible based on accumulated knowledge about gender norms in our society. First, men, driven by perceptions of masculinity, are generally less inclined to report their symptoms and seek health care assistance than women [32]. Moreover, they are more inclined to report good health conditions. Second, women’s health outcomes are worse than men’s health outcomes because they are burdened by the double role they play when integrated into the workforce, combining caregiving responsibilities for children and family members, household chores, and paid work [17]. These role dynamics may explain the higher prevalence of negative SRH among women, as their margins for recovery from the effects of paid work are narrow. In other words, sleep duration is shortened, and time is restricted for leisure activities and other healthy behaviors, among other consequences that lead to symptoms and morbidities [33]. Third, due to household responsibilities and conditions surrounding motherhood, women are driven to seek part-time jobs, which tend to offer lower remuneration compared to regular working hours [34]. Financial constraints reduce access to essential resources for good health [17, 35].

Male and female workers who were engaged in informal employment had a higher likelihood of reporting negative SRH. This result was expected [36, 37]. First, in informal employment, the employer, by not declaring the employment relationship, avoids social security expenses and taxes. As a consequence, employees’ access to social security benefits is restricted, meaning that they will not be covered by benefits when they are absent from work due to illness or disability. Second, in informal labor market environments, the conditions are more harmful because they are generally not covered by existing regulations that limit exposure to toxic agents and other protective norms [38, 39].

In the specific case of Brazil, as well as in other low- and middle-income countries, the proportion of the workforce in the informal sector is increasing where lower wages, atypical working hours, and short-term contracts prevail over long-term contracts [40]. Jobs with such characteristics are primarily occupied by women, older individuals, nonwhite individuals, and those with lower levels of education, reflecting the structural inequality in the Brazilian labor market [28, 40, 41]. In summary, informal employment is an indicator of social inequalities with known effects on workers’ health [36–38, 42, 43].

Converging with our results, the study by Utzet et al. in 2021, which examined the association between SRH and informality in Latin American and Caribbean countries by gender, revealed a worse health situation among informal workers. However, it did not identify significant differences between men and women in this context [43]. Perhaps in the current scenario of changing work dynamics [44], the impact of exposure to adverse working conditions and lack of social protection, characteristic of informal employment, did not produce specific sex/gender effects on health [45]. Alternatively, this result might conceal uninvestigated situations, such as the number of children, for example.

However, despite women having a higher level of education, no significant differences were observed regarding per capita income, employment status, age group, or skin color/race among the group of workers with prolonged working hours for both men and women (Table 2). These results suggest a similarity in the sociodemographic profiles of the workers. If this is the case, the observed outcomes may have been biased.

Possible biases due to the criteria used to define employment status in the NHS should be acknowledged. It is also worth noting that in Brazil, the labor law of 2017 introduced several changes in employment relationships, including the flexibilization of labor regulations and the possibility of less protected employment contracts [6]. It is possible that changes in legislation have reduced differences in working conditions between men and women, which, as mentioned above, could have affected SRH. In addition to the new regulations, novel forms of work, such as remote work and app-based work, have introduced organizational arrangements that have not yet been well evaluated or assessed in terms of occupational exposure, workload, and health perception [46].

The use of self-reported data and the lack of more detailed information on working conditions, such as exposure to specific risks or the intensity of work demands, are limitations to consider in interpreting the results, in addition to those mentioned above. Although statistical care and adjustments for relevant confounding variables were adopted, the role of unconsidered modifiers, such as support networks, cannot be excluded. Longitudinal designs are desirable for further investigating the relationship among working hours, employment status, and workers’ health, considering other factors such as gender power dynamics.

The strengths of this study deserve recognition. The representativeness of the NHS data for the national territory fills gaps previously observed in studies on workers’ health, such as homogeneous samples or surveys that did not distinguish employment contracts [47]. SRH is a recognized and widely used health indicator [21]. An association between negative health and prolonged working hours was found in both men and women. This result provides strong arguments for discussions on the harms of labor deregulation in Brazil [28].

The results provide clues for public health actions and occupational health promotion programs, aiming to bring the effects of gender norms in our society into the decision-making arena at the macrostructural level. Intersectoral actions (employment, health, economic development) would be appropriate for defining temporal organization modalities of working hours. Concerted efforts among governments, employers, and health professionals would be useful for developing guidelines to monitor the damages to informal employment. Actions in this direction would be fundamental steps toward reducing social health inequalities.

## Conclusion

In conclusion, working hours and employment status were independently associated with SRH. We found a significant interaction between long working hours and informal employment in relation to negative SRH. Gender differences in the results were observed when considering differences in working hours, supporting our initial hypothesis. Among those working long hours, women have a higher prevalence of negative SRH. Particularly noteworthy are female informal workers engaged in long working hours, who exhibited a higher probability of negative SRH among the analyzed profiles. These findings underscore the importance of addressing gender differences in occupational health research. Our study was an attempt to contribute to the literature by addressing this issue in working populations in low- and middle-income countries.

## Data Availability

The dataset analyzed during the current study is available in the repository of the Brazilian Institute of Geography and Statistics (IBGE) (https://www.ibge.gov.br/estatisticas/sociais/saude/9160-pesquisa-nacional-de-saude.html). The data are publicly available.

## Abbreviations

IBGE: Brazilian Institute of Geography and Statistics
NHS: National Health Survey
SRH: Self-rated health
GDP: gross domestic product
CI: confidence intervals
OR: odds ratio
AOR: adjusted odds ratio
